# Triad of Idiopathic Thrombocytopenic Purpura, Preeclampsia, and HELLP Syndrome in a Parturient: A Rare Confrontation to the Anesthetist

**DOI:** 10.1155/2014/139694

**Published:** 2014-12-08

**Authors:** Tanu Mehta, Geeta P. Parikh, Veena R. Shah

**Affiliations:** Department of Anaesthesia and Critical Care, Smt. K. M. Mehta and Smt. G. R. Doshi Institute of Kidney Diseases and Research Center, Dr. H. L. Trivedi Institute of Transplantation Sciences, Civil Hospital Campus, Asarwa, Ahmedabad, Gujarat 380016, India

## Abstract

Idiopathic thrombocytopenic purpura (ITP) with HELLP represents a rare complication that requires combined care of obstetrician, anesthesiologist, hematologist, and neonatologist. At 37-week gestation a 35-year-old parturient (G2A1P0) a known case of chronic ITP presented with severe pregnancy induced hypertension (PIH), thrombocytopenia, and elevated liver enzymes. We describe successful anesthetic management of this patient who was taken for emergency caesarean section.

## 1. Introduction 

The concomitance of chronic ITP, preeclampsia-eclampsia syndrome, and HELLP syndrome in a parturient is not very well described in literature. In January 2010, Ben et al. did a bibliographical search in PubMed and Cochrane database of systematic reviews and no similar cases were reported [1]. We describe the anesthetic management of a patient of chronic ITP who develops preeclampsia syndrome with HELLP syndrome.

## 2. Case Report 

A 35-year-old parturient (G2A1P0) at 37 weeks of gestation presented at emergency department with severe preeclampsia. An urgent lower segment caesarean section (LSCS) was planned by the obstetric team and anaesthesia consultation was sought for it. Her antenatal history revealed that she was a patient of chronic ITP which was diagnosed 1 year back when she developed menorrhagia after diagnostic hysterolaparoscopy. She had thrombocytopenia and her bone marrow biopsy revealed normocellular marrow with megakaryocytes. She received methylprednisolone (20 mg/day) for 6 months which was later tapered down. She was on regular antenatal care (ANC) and did not receive any treatment during pregnancy as she had mild thrombocytopenia and her platelets remained around 100–150 × 10^9^/L. In her last ANC which was 5 days prior to admission her platelets were normal and BP was 130/80 mm of Hg. On admission she had c/o headache and blurring of vision with severe epigastric pain and vomiting. Her BP was 210/110 mm Hg and was treated with sublingual depin 10 mg and loading dose of Inj. MgSO_4_ followed by 5 gm IM in each buttock alternately. Inj. dexona 2 mg IV was given. Since her liver enzymes were elevated (SGPT 422 IU/L, SGOT 765 IU/L, S. Bilirubin total 3.6, direct 2.4, and indirect 1.2) HELLP syndrome was suspected. CBC showed normal S. creatinine (0.7) and electrolytes (Na, K, Ca, and Mg). Her Hb was 9 gms%. Platelet count was 60 × 10^9^/L, LDH was 958 IU/L, and INR was normal. Blood products including platelet concentrates were kept available. She was transported to the OT with a wedge under right hip. Premedication included IV ranitidine (150 mg) and metoclopramide (10 mg). Following adequate preoxygenation with 100% O_2_, induction of anesthesia was achieved with thiopentone sodium 300 mg (5 mg/kg). Aided with Sellicks manoeuvre, tracheal intubation was done with Portex Cuffed Endotracheal Tube number 7.0 after adequate relaxation with succinylcholine (2 mg/kg). Anesthesia was maintained with sevoflurane before and nitrous oxide after the delivery of baby and bolus doses of atracurium were given as muscle relaxant. IV fentanyl 100 *μ*g was given after the baby was delivered. Intraoperative monitoring included temperature, pulse, BP, SpO_2_, ECG, and etCO_2_. A healthy female child was delivered 16 minutes after incision and APGAR scores were 7 and 9 at 1 and 5 minutes, respectively. Five minutes after baby delivery BP went up to 180/100 mm of Hg and NTG infusion was started. Inj. oxytocin 20 U in IV infusion was started and Inj. prostodin 250 mg IM was given immediately after the baby's delivery. Surprisingly there were no problems of haemostasis and the patient received 1.5 litres of crystalloids. She was reversed with neostigmine (2.5 mg) and glycopyrrolate (0.2) and shifted to ICU for postoperative care. IV tramadol (2 mg/kg) was given for postoperative analgesia. In the first two postoperative days platelets went down to 25 × 10^9^/L and LDH increased to 1592 IU/L. From the 3rd postoperative day platelet counts started improving with a decreasing trend of liver enzymes and LDH. The patient was discharged on 9th postoperative day with normal liver enzymes and platelets and LDH was down to 493 IU/L. There was no evidence of neonatal thrombocytopenia.

## 3. Discussion 

ITP is present in 0.01-0.02% of women during pregnancy. The decreased platelet count is due to binding of autoantibodies directed against target antigens on platelets, specifically the glycoprotein IIb-IIIa complex or glycoprotein Ib. These antibodies serve as opsonins accelerating platelet clearance by phagocytic cells in the reticuloendothelial system [2]. Management of a pregnant woman with ITP is based on the assessment of the risk of significant hemorrhage. The platelet count usually falls as pregnancy progresses with the greatest rate of decline occurring in the 3rd trimester [3]. Frequent monitoring of platelet count is required to ensure a safe platelet count at the time of delivery. In our patient platelets remained normal throughout the pregnancy and the first episode of thrombocytopenia was at the time of admission. Severe preeclampsia was diagnosed as the patient had hypertension with epigastric pain (stretching of liver capsule) and headache (sign of cerebral edema) and urine albumin was +2 by dip stick test. Elevated liver enzymes and S. Bilirubin and raised LDH correspond to the diagnosis of HELLP syndrome. As a part of preanaesthetic evaluation strategic planning is essential for anticipating difficulties like hemorrhage in the respiratory tract, aspiration of gastric contents, uncontrolled hypertension, convulsion, and fetal hypoxia and controlling these problems promptly. ITP itself is not an indication for caesarean delivery or general anesthesia. Mode of delivery and anesthesia in a pregnant patient with ITP is based on obstetric indication with avoidance of procedures associated with increased hemorrhagic risk. Maternal anesthesia must be based on safety of the mother. The HELLP syndrome affects 10–20% of women with severe preeclampsia but 15–20% of women do not have antecedent hypertension or proteinuria and symptoms may be absent [4]. However, since our patient was already symptomatic and signs of haemolysis were present (increased LDH) we decided to give her general anesthesia (GA). In addition to the usual precautions of GA in pregnancy there are special problems that increase the anesthetic risk associated with such concomitant conditions. These include edema of upper airway, severe hypertensive response to laryngoscopy, interaction of magnesium with muscle relaxants, and risk of haemorrhage. Preoperative medication, Sellicks manoeuvre, and rapid sequence induction were used to avoid Mendelson's syndrome. Another therapeutic dilemma was the issue of platelet transfusion. Patients with ITP are generally refractory to platelet transfusion because of platelet alloantibodies. Conventionally a platelet count of ≥50 × 10^9^/L should target transfusion count and unnecessary transfusion of platelet concentrates in the absence of haemostatic failure may stimulate more autoantibodies and worsen maternal thrombocytopenia [2]. Low platelets in our patient were more likely due to HELLP and not ITP as she was in remission; however, we did not transfuse any platelets as her preoperative platelet count was 60 × 10^9^/L and there was no problem with haemostasis. Risk of hemorrhage in respiratory tract due to thrombocytopenia and compromised airway was avoided by treatment with smooth, rapid, and atraumatic intubation with the help of small endotracheal tube, adequate expertise, and better muscle relaxation with succinylcholine. Hypertensive response to laryngoscopy was attenuated with IV lignocaine. Intensive postpartum monitoring is necessary in women with HELLP because laboratory parameters frequently worsen 24–48 hours after delivery with peak rise in LDH and platelet nadir and platelet count begins to rise by 4th day postpartum [5] as was the case in our patient where parameters started improving by day 3. ITP is characterized by a reduced platelet count and increased peripheral destruction of platelets and augmented platelet production was evidenced by increased circulating megathrombocytes and higher number of megakaryocytes in bone marrow. Preeclamptic individuals have similar antiplatelet antibody profiles. The disorder differs in that the neonatal platelet counts are not depressed in preeclampsia whereas in ITP neonatal thrombocytopenia results from placentally transmitted maternal antibody [6]. Therefore platelet count of the neonate should be obtained at delivery to determine the need for immediate therapy. In neonates with thrombocytopenia platelet count is obtained daily because its nadir is frequently seen in 2–5 days after birth. A spontaneous rise of platelet count is usually seen by day 7. There was no evidence of neonatal thrombocytopenia in our patient. Another rare differential diagnosis to HELLP is TTP (thrombotic thrombocytopenic purpura). TTP represents a rare complication mainly in the second trimester and mimics HELLP syndrome in its early stage. Patients with HELLP syndrome completely recover following delivery. In contrast TTP does not usually improve dramatically following delivery or monotherapy with corticosteroids. Also, if LDH to AST ratio exceeds 25 : 1, especially in association with severe haematuria with failure of the platelet count and LDH to respond to therapy, a presumptive diagnosis of TTP can be considered and emergent plasma exchange (PEX) needs to be initiated [6]. However, since the patient improved after caesarean section and LDH to AST ratio was low [7] it further corresponded to a diagnosis of PIH with HELLP in a patient of ITP.

## 4. Conclusion 

In summary, a high level of vigilance and team work is necessary in a parturient with ITP. Medical and expectant management can suddenly change into surgical and anesthetic management if patient develops HELLP syndrome. Establishment of an accurate diagnosis and individualized management is required to obtain best outcome in this clinically diverse condition.
